# Effects of Resistance Training on Skin Temperature and Its Relationship with Central Nervous System (CNS) Activation

**DOI:** 10.3390/healthcare10020207

**Published:** 2022-01-21

**Authors:** Manuel Sillero-Quintana, Jacob Jones-Rando, Ignacio Refoyo, João Carlos Bouzas Marins, Adérito Seixas

**Affiliations:** 1 Faculty of Physical Activity and Sport Sciences (INEF Madrid), Universidad Politécnica de Madrid, 28040 Madrid, Spain; jacobjones200m@gmail.com; 2 Department of Physical Education, Federal University of Viçosa, Viçosa 36570-900, Brazil; jcbouzas@ufv.br; 3 Escola Superior de Saúde, Fundação Fernando Pessoa, 4249-004 Porto, Portugal; aderito@ufp.edu.pt

**Keywords:** infrared thermography, sympathetic activation, strength training

## Abstract

The aim of this work was to relate the activation of the sympathetic and parasympathetic nervous systems with the skin temperature (Tsk) of the lower limbs after a resistance training exercise. Under controlled conditions, the average Tsk in the areas of the anterior and posterior thighs, knees and legs was obtained with a thermal imager and the parasympathetic and sympathetic activation was registered with an Omegawave^®^ device on 20 healthy and trained male volunteers (25.39 ± 8.21 years) before exercise, immediately after standard resistance training (3 exercises (2 quadriceps + 1 hamstrings) × 4 sets × 10 repetitions (70% 1RM), 90-sec recovery) and after 20 min of recovery. The results showed a significant effect of exercise and recovery on Tsk in all regions of interest (ROIs) considered (*p* < 0.05) and strong inverse relationships between sympathetic and parasympathetic activation values. Significant results were found for the total variation of Tsk (*p* < 0.05) with highly positive values for subjects with lower sympathetic activation and almost null or even negative values for those with higher sympathetic activation. Sympathetic activity was a significant predictor of total Tsk variation in the anterior thigh, posterior thigh and anterior knee but not in the posterior knee, anterior leg, and posterior leg. Baseline Tsk was a significant predictor of total Tsk variation the all ROIs except in the posterior knee. Tsk measured by thermography could be used to estimate the level of participation of muscle areas in exercise and registering the level of sympathetic activation before exercise could be interesting in predicting the athlete’s physiological response to strength training.

## 1. Introduction

Infrared thermography (IRT) is making its way into the world of sport, especially in the field of injury prevention [1,2]. Thermographic cameras capture the infrared radiation of the electromagnetic spectrum, converting the radiated energy into information about the superficial skin temperature (Tsk), which is directly related to the local blood flow of the skin. There are many medical applications of IRT [3], but some researchers have shown new applications for thermography in the sports area [2], such as quantification of training load [4], detection of biomechanical imbalances [5], injury prevention [1,6] and the effects of the sports practice on the athlete’s skin temperature [7,8,9].

Considering the last application mentioned above, it has been reported that highly trained subjects have a lower Tsk during exercise and need a longer time to recover the baseline Tsk after exercise [5,9]. After exercise, metabolic activity remains increased as a compensatory response in order to recover after the catabolic period. Therefore, athletes may need a longer resting time, being imperative to review their diets, hours of sleep and recovery protocols [2]. 

The body’s thermoregulation system involves many factors and processes to maintain homeostasis. During exercise, homeostasis in general, and Tsk in particular, is strongly affected by skeletal muscle (metabolism), the cardiovascular system (blood flow) and the nervous system (central and local) [10], and it is also influenced by the type of exercise [11], its duration and intensity [12,13], and the environmental conditions under which the exercise is performed.

Direct current potential (DC potential) has been used to measure brain function and is a great indicator of the functional state of the Central Nervous System (CNS) and the level of subject functionality [14]. There is not much information on the DC potential and sports, primarily due to the fact that most studies were carried out in the Soviet Union and they have not yet been translated, and, secondly, because there is no standard terminology; therefore, researchers have given different names to the term ‘DC potential’, for example, slow cortical potential [15], steady potential [16], or infra-slow cortical potential [17]. Omegawave^®^ is a tool that is currently used not only in the medical area [18,19], but also in sports in which it has been validated [20], where coaches and sport scientists use it to understand how athletes’ bodies work and to improve their fitness in the different stages of the training process, using variables related to brain activation and heart variability, providing information about the level of sympathetic and parasympathetic activation of athletes [21,22].

Due to the lack of information on the issue, the aim of this exploratory study was to investigate the relationship between the activation of the sympathetic and parasympathetic systems and the skin thermal response of athletes measured by IRT immediately after strength training and after 20 min of recovery.

## 2. Materials and Methods

### 2.1. Definition of the Sample

A sample size calculation was initially performed with G*Power software (Heinrich-Heine-Universität, Düsseldorf, Germany). The effect size of exercise on skin temperature changes reported in the literature is large [23,24], considering an effect size of 0.4, a statistical power of 0.80, and three repeated measurements for the repeated measures ANOVA assessing the effects of resistance training on skin temperature, a minimum sample size of 12 was calculated. To account for any dropouts and other unforeseen shortcomings, 20 healthy and trained male volunteers aged 25.39 ± 8.21 years (height: 178.88 ± 8.37 cm; weight: 82.38 ± 6.80 kg; BMI: 25.76 ± 1.45) were recruited. 

Each participant signed their informed consent before starting the data collection session. They were highly trained sport sciences students and athletes from the Spanish high-performance training center of Madrid, who trained 12.25 ± 4.19 h a week and with a training experience of 10.25 ± 4.92 years. All of them were highly skilled at resistance training and reported a healthy condition without any disease the previous week of the data collection moment. 

The study began after the approval of the protocol by the Ethics and Research Committee of the UPM (THERMOSPEC project, PIA 12009-11) and all procedures were carried out in accordance with the Helsinki recommendations [25] and Spanish regulations regarding human studies.

### 2.2. General Aspects of the Data Collection Procedure

**Figure 1:**
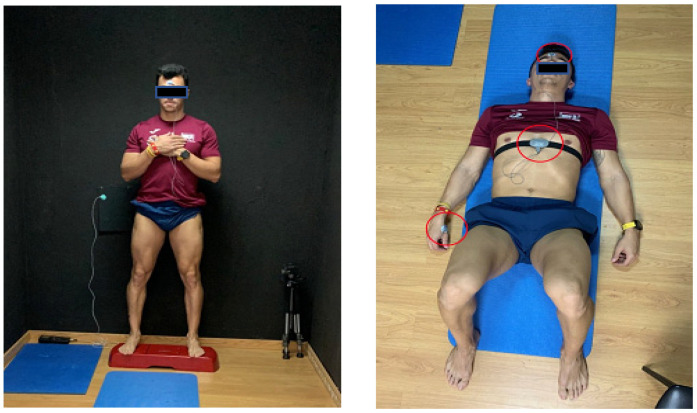
Images of the data collection protocol.

Data collection was performed in a thermography data collection room, equipped with special technology required for the study, which was 10 m away from the gym where the training sessions were performed. The thermography room had a non-reflective black painted wall, which was used as a background for better performance and precision of thermal data collection (Figure 1). It was equipped with an air conditioning system to control the ambient conditions of the room and with a BAR-908-HG^®^ weather station (Oregon Scientific, Portland, OR, USA) to record the temperature and humidity of the room during data collection. The ambient conditions were quite stable during the entire data collection period (room temperature: 18.6 ± 1.3 °C; humidity: 41.8 ± 6.5%). 

### 2.3. Description of the CNS Activation Assessment

Omegawave^®^ equipment (Omegawave Technologies, Portland, OR, USA) was used to determine the level of CNS activation. The Omegawave^®^ system consists of an ECG strap with an Omegawave sensor attached to it, measurement cables and electrodes for DC measurements (Figure 1), and the Omegawave app installed on an iPad. This device records different variables from the ECG and DC potential of the brain, which are related to the variability of heart rate and brain activation, including the time domain parameters (SDNN, SDSD, and RMSSD) that reflect the status of the autonomic nervous system, frequency parameters (total power, LF/HF, HF, normalized HF, LF, and normalized LF) related to the status of the autonomic nervous system, and DC potentials [26]. These variables are integrated by Omegawave^®^ to determine the level of activation of the parasympathetic and sympathetic autonomous nervous system with an index between 0 and 1.

A 12-cm-high plastic step was used to isolate subjects from the ground during thermographic data collection, and a training mat allowed participants to lay down comfortably during Omegawave^®^ assessment, thus avoiding contact with the ground.

Due to the characteristics of the study, the original Omegawave^®^ protocol was slightly modified: the subject laid on his back but with his legs 90° flexed, in order to avoid contact of the posterior part of his lower limbs on the mat to not interfere with thermographic results, due to the contact of the skin on the mat surface and to allow normal convection on the skin mainly during recovery after strength training.

### 2.4. Description of the Skin Temperature Assessment

The thermographic camera used was an FLIR T530 (FLIR Systems, Stockholm, Sweden) with a sensor array size of 320 × 240 pixels, thermal sensitivity (NETD) < 40 mK, accuracy of ±2% of the overall reading, spectral range of 7.5 to 14.0 µm. The emissivity of the camera was set to 0.98. The camera was turned on at least 10 min before starting the data collection in order to stabilize the sensors, being placed perpendicular to the subject at a distance of 3 m, avoiding interferences with any source of infrared radiation. 

Data collection was carried out following the guidelines of the TermoINEF protocol [27] and the recommendations of the TISEM consensus document [28]. 

### 2.5. Description of the Resistance Training

**Figure 2:**
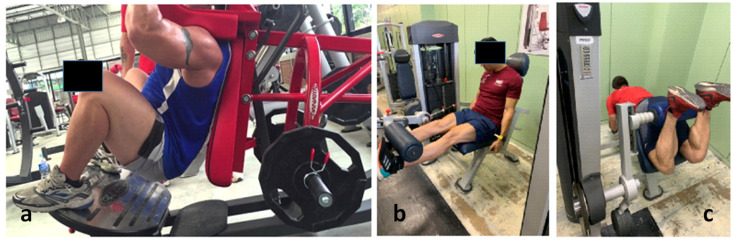
Exercises included in training (**a**) Power Squad, (**b**) Knee extension, and (**c**) Hamstring curl (prone femoral curl).

Resistance training consisted of a 5-min progressive warm-up protocol on a cycle ergometer, 5 min of joint mobility and a set of 4–5 repetitions with a minimum load on the three exercises (Figure 2) to make the last adjustments in the machines and finish the warm-up. Once the warm-up was completed, the subject was asked to begin their training consisting of three lower body exercises (one focused on hamstrings and two on quadriceps) performed on Panatta^®^ equipment (Panatta, Apiro, Italy). Power squat, knee extension, and prone femoral curl (Figure 2a–c). Subjects performed 4 sets of 10 repetitions (1:1;—1″ eccentric and 1″ concentric—at normal speed, with the total movement range of the machine and an angle of knee flexion lower than 90° in all the cases) of each exercise at 70% of their 1RM, which was estimated immediately after the warm up and before training by Bryzcki’s equation [29] after performing a set of the maximum number of repetitions with a known load. Recovery between sets and exercises was 90 s.

### 2.6. Data Collection Procedure

**Figure 3:**
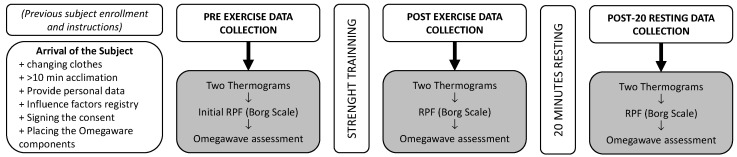
Structure of the data collection procedure.

Figure 3 summarizes the data collection procedure. Before the data collection day, subjects were instructed to avoid physical activity at least 12 h before data collection, sleep at least 8 h, arrive at the laboratory by private car or public transport, and bring shorts and training shoes to allow a correct training session and data collection.

After arriving in the data collection laboratory (between 8:30 and 13:00 h), the subjects changed their clothes for shorts and acclimatized to the room for a minimum period of 10 min, while signing the informed consent document of voluntary participation in the study and providing their personal data and other variables that can influence the thermography or be useful for data use (for example, the analysis of the data, such as means of transport, consumption of substances such as coffee or drugs that could affect data collection, or moment of the last intense physical activity) [30].

After fulfilling the questionnaire, the subject was prepared for Omegawave^®^ assessment, placing the chest strap of the ECG with the BLE sensor and electrodes on the forehead and dominant hand (see red circles in Figure 1), following the manufacturer’s instructions. 

Subsequently, the subjects stood on the step, and two thermograms of the anterior and posterior lower extremities of the subjects were recorded and the perceived exertion rate (RPE) was estimated using the 10-points Borg scale [31], with an initial baseline score of 3.10 ± 2.13.

Finally, subjects were asked to lie on the mat on their back and relax for around 3–4 min to perform the Omegawave^®^ assessment. 

Immediately after the strength training, the participant returned to the thermography data collection room for a second set of thermograms (anterior and posterior), RPE score (6.75 ± 1.18) and Omegawave^®^ assessment.

Considering that the acute effects of the exercise on skin thermal response are different even after a short recovery time [23], a last set of thermograms, RPE scores (5.26 ± 1.81) and Omegawave^®^ were taken 20 min after completion of strength training. During this resting time, the subject remained seated on their gluteus, avoiding any contact of the posterior thigh with the chair to prevent any interference with the Tsk assessment. 

### 2.7. Analysis of the Data

**Figure 4:**
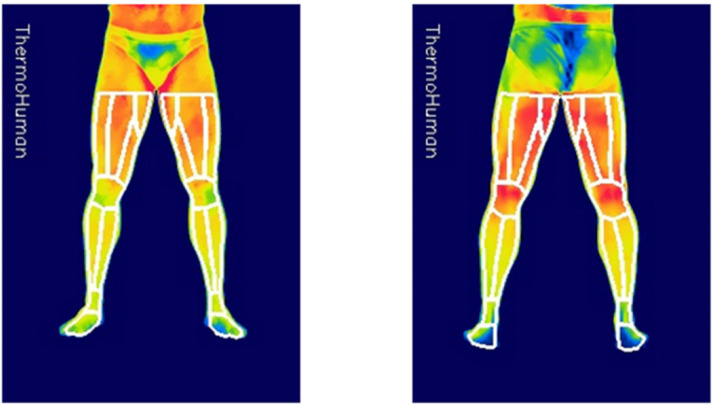
Thermograms included in Thermohuman^®^ software and an example of ROI selection in the posterior view.

In order to analyse the thermograms, the software Thermohuman^®^ 2.0 (Thermohuman, Madrid, Spain) was used for the extraction of Tsk data from the thermograms. This software automatically delimits 36 regions of interest (ROIs) from the two registered thermograms (i.e., anterior and posterior lower limbs) by using automatized artificial vision, providing average, maximum, and minimum Tsk of each ROI, its standard deviations, and number of pixels (Figure 4).

All data were collected at three moments: before exercise (PRE), immediately after exercise (POST), and after 20 min of passive recovery (POST-20). The average time between the PRE and POST thermographic data collection (‘exercise’ duration) was 51 ± 6 min and between POST and POST-20 (‘recovery’ duration) was 20 ± 4 min, which is the total data collection time of 71 ± 5 min.

As the 36 ROIs provided by Thermohuman^®^ would generate too many data for further analyses, those corresponding to feet and ankles were discarded and, considering that the level of asymmetry between contralateral regions was lower than 0.5 °C, the rest of the ROIs were integrated into six wider body regions including the values of the right and left side on them (i.e., Anterior and Posterior: Thigh, Knee, and Leg) by averaging the Tsk values using the number of pixels of each ROI obtained by Thermohuman^®^. Variations of Tsk for the three evaluation moments were included in statistical analyses (Δ-exercise = Tsk POST- Tsk PRE; Δ-recovery = Tsk POST-20−Tsk POST; Δ-total = Tsk POST-20−Tsk PRE).

Omegawave^®^ provides a wide list of variables corresponding to heart rate variability and central nervous system activation (DC potential). For this study only parasympathetic activity (values rated (0–1)); and sympathetic activity (values (0–1)) were considered.

Finally, to study the level of influence of the CNS activation level on the thermal response to resistance training, the whole sample was divided into two groups considering as cut-off point the median (0.53) of the initial value of sympathetic activation (Lower activation: ≤0.53; *n* = 10; Higher activation: >0.54; *n* = 10).

The Statistical Package for the Social Sciences software (SPSS Statistics version 26, IBM Corp., Armonk, NY, USA) was used to perform the statistical analysis, setting the level of statistical significance at *α* = 0.05.

After applying the Kolmogorov–Smirnov normality test to all the considered variables, it was found that the distribution of the data was normal; so that means and standard deviations were employed to explain the general results. After checking for assumptions regarding sphericity violations, repeated measures ANOVA were performed on the Tsk average values by moment (PRE-, POST- and POST-20) for each considered ROI, using Bonferroni post-hoc analysis to determine significant differences between moments. Partial eta-squared estimates (η²_p_) were used to assess effect size. 

To understand the relationship between sympathetic and parasympathetic activation levels at the three considered moments, Spearman’s correlations were calculated. Mann–Whitney U and Student’s t tests were performed for variables without normal distribution and variables with normal distribution, respectively, to determine the influence of the level of sympathetic activation on variations of Tsk (ΔTsk) after the resistance training. To estimate the confidence intervals between groups, the Hodges–Lehmann estimation and mean differences were used for variables without normal distribution and variables with normal distribution, respectively, and to estimate the effect size, the rank biserial correlation and Cohen’s d were used for variables without normal distribution and variables with normal distribution, respectively. 

Linear regression models for each ROI (method enter) were tested, considering total (Tsk POST-20−Tsk PRE) as the dependent variable and sympathetic activity, adjusting for baseline skin temperature in the ROI as independent variables. The assumptions of the models were assessed graphically (residual distribution and homoscedasticity) and through the Durbin–Watson statistic. The multicollinearity between the independent variables was assessed using the variance inflation factor. The assumptions and multicollinearity analysis did not reveal relevant issues.

## 3. Results

Table 1 summarizes the Tsk values together with the levels of sympathetic and parasympathetic activation at the three different moments of data collection and the results of a repeated measures ANOVA analysis for the Tsk values over time (PRE, POST, and POST-20).

There was a significant effect of time on the Tsk in all ROIs with large effect sizes. Figure 4 summarizes the variations of Tsk between the three considered moments (Δ-exercise, Δ-recovery, and Δ-total), indicating the significance of the variations according to the Bonferroni post-hoc analysis. However, the variation of the sympathetic and parasympathetic activation levels does not show significant differences over time. It is worth noting the high values of the standard deviations both in the case of sympathetic and parasympathetic activation, with values ranging from one third to one half of the mean values of these variables.

Table 2 summarizes the Spearman correlation analysis and shows strong inverse relationships between the values of sympathetic and parasympathetic at the same data collection moment and also between the moments of POST and POST-20, but not between the moments PRE compared with POST and POST-20. 

This led us to consider only the sympathetic activation level to divide the sample into two groups (“lower” and “higher” sympathetic activity). Variations of Tsk for the two groups at the three considered moments (“Δ-exercise”: POST−PRE; “Δ-recovery”: POST-20−POST; and “Δ-total”: POST-20−PRE) and the results of the parametric (Student’s T test) and non-parametric (U of Mann–Whitney) analysis can be seen in Table 3.

Regression analysis showed that the models were significant for all the ROIs considered, but the total variance explained by the models (adjusted R2) ranged between 23.0% in the initial Tsk of the posterior knee and 63.7% and 64.4% in the initial Tsk of the anterior thigh and posterior thigh. Sympathetic activity was a significant predictor of the Δ-total of Tsk in the anterior thigh, posterior thigh and anterior knee, but not in the posterior knee, anterior leg, and posterior leg. Baseline Tsk was a significant predictor of Δ-total of Tsk for all ROIs, except in the posterior knee (Table 4). 

## 4. Discussion

The results of this work could explain the acute and short-term effects of resistance training on the Tsk of the lower extremities, the balance between the levels of sympathetic and parasympathetic activation in the different phases of data collection, and the influence of the level of sympathetic activation on the variations of Tsk after training and a 20-min recovery.

**Figure 5:**
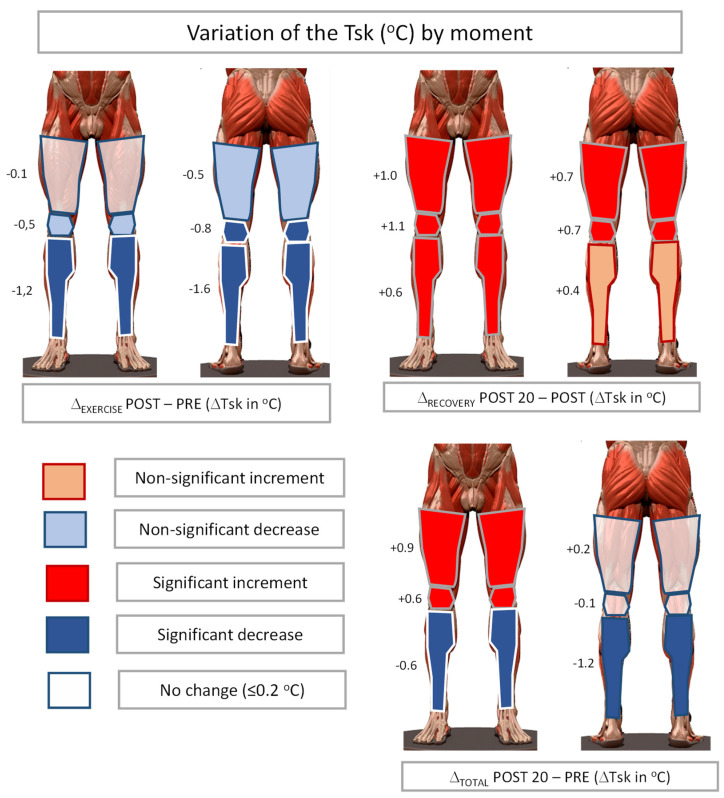
Variation of Tsk between the three recorded moments according to the Bonferroni post hoc test.

Table 1 summarizes the results of a repeated measures ANOVA analysis for the Tsk values over time (PRE, POST, and POST-20), and clearly shows that resistance training generates significant Tsk variations on the ROIs considered. This effect can be easily visualized in Figure 5, with a significant decrease in Tsk immediately after exercise in areas with lower muscle activity during exercise, reaching −1.6 °C in the posterior legs, and a lack of variation in the anterior thigh (i.e., quadriceps), which was the most stressed area with two exercises performed, and non-significant descents of −0.5 °C in ROIs that were activated during the programed exercises but with lower intensity than the quadriceps muscle (i.e., anterior knee corresponding to the quadriceps’ tendon and patella, and posterior thigh that only performed one exercise). 

These results are similar to those obtained by Fernandez Cuevas and his collaborators [11], who registered quite variable ROIs corresponding to joints, pronounced decreases up to −1.8 °C in non-exercised areas, and almost null variations in exercised areas of the anterior thigh immediately after a quadriceps strength training protocol. The reduction in Tsk values in non-exercised areas after resistance training could be explained by a redistribution of blood flow from the skin to the muscles activated during exercise [32]. Considering that exercised areas produce heat and require more blood flow to provide nutrients and reduce the temperature of active muscles [33], it seems logical that, after resistance training, the Tsk of exercise areas behaves differently from the non-exercised areas, with an indirect relationship of Tsk response according to workload, the lower the Tsk decrease in areas where muscle workload is higher.

Another factor that could contribute to the acute drop in Tsk immediately after exercise could be sweat evaporation that occurs due to increased metabolic activity during exercise, which causes the internal temperature to rise, producing sweat to reduce it at the skin level. However, it should be noted that, under neutral environmental conditions and considering the short duration of the effort, the influence of this factor does not seem very relevant in describing the reduction of Tsk.

Interestingly, when the results of Tsk after 20 min of recovery were considered, our results show a generalized significant increase in Tsk in the lower extremities except for the posterior legs, the Tsk values being higher in the exercise areas. It is well known that when exercise is finished, blood flow and sweat rate increase on the skin to facilitate post-exertional heat dissipation [34,35]. It seems that the modification of the cutaneous vascular conductance in the exercised areas could be wider to dissipate the excess heat produced in the muscle. This would allow more warm blood to reach the skin in these areas, leading to a higher elevation of Tsk.

Finally, the ‘total’ results of Figure 4 integrate the responses ‘exercise’ and ‘recovery’, with a significant increase of +0.9 °C in the most exercised areas, a lack of variation of Tsk in the less exercised area (i.e., posterior thigh) and a reduction of Tsk in the non-exercised areas. It is important to note that due to the different thermal responses to the exercise according to the moment of data collection, time elapsed since the end of the exercise should be considered to evaluate the effects of the exercise by IRT and to compare the results of different studies using infrared cameras to evaluate the Tsk response to the exercise. 

Table 1 summarizes the evolution of the sympathetic and parasympathetic activation level at the three data collection moments. Even when strength exercise generated the typical increase in sympathetic activation [12] and a subsequent reduction in parasympathetic activation, the high standard deviation values of the mean values for the baseline and after the exercise results indicate a high variability of the initial values and responses of the participants to the exercise. 

Even when the results of the repeated measures analysis were not significant, the results of the Spearman correlation analyses reflect a strong inverse relationship between values of sympathetic and parasympathetic at the same data collection moment. The averaged values of sympathetic and parasympathetic activation were 0.55 and 0.42, respectively, before exercise, and changed to 0.61 and 0.36 immediately after exercise. Resistance training seems to increase sympathetic activity and decrease parasympathetic activity, reactions that were explained by Kingsley and Figueroa [35], who stated that post-exercise muscle ischemia increases vascular sympathetic modulation through baroreflex activation induced by local metabolites in the exercised muscle, and elevated blood pressure reactivates baroreflex sensitivity and cardiac parasympathetic modulation during this condition. After recovery, our results show values of 0.59 for sympathetic and 0.39 for parasympathetic activation, which indicates that the baseline levels of activation had not yet been completely restored.

The strong and inverse relationship between sympathetic and parasympathetic activation led to the division of the sample into two groups (“lower” and “higher”) to search for the influence of previous level of sympathetic activation on the variation of Tsk after exercise and after recovery. In Table 3, it can be seen that, probably due to the high variability of the results, even when the differences between activation groups for the variations of Tsk after exercise were not significant, the most activated muscle area (that is, the anterior thigh) was the single ROI with a positive variation of Tsk after exercise, but only in the case of the lower sympathetic activation group (+0.29 °C) and for most of the considered ROIs, except for the Tsk anterior knee, the variations Tsk immediately after exercise area for the lower sympathetic activation group were lower than for the higher sympathetic activation group. It seems that subjects with low sympathetic activation could be in a different vascular condition before starting exercise, and the effects of sympathetic activation generated by exercise would be stronger for this group. 

Taking into account the variations of Tsk after 20 min of recovery, it can be seen that the variations of the most exercised areas (i.e., Anterior Thigh and Knee) were higher in the subjects of the lower activation group (1.07 and 1.38 °C for Lower vs 0.52 and 0.54 °C for Higher) but once more, these differences did not reach statistical significance, probably due to the high variability of the results.

However, considering the results of the total Tsk variation, we found statistically significant results, obtaining higher Tsk variations for the “lower” activation group in the most activated regions (i.e., anterior thigh and knee and posterior thigh), the variation being highly positive for the “lower” activation group and almost null or even negative for the “higher” activation group. This fact could be important to understand the different inter- and intra-individual Tsk responses to exercise and the high variability of the responses to exercise in humans, which are even related to variations in the thermal pattern [36], because these different Tsk responses to exercise could be related to previous neural activation levels of subjects before starting data collection. 

In fact, the regression analysis in Table 4 confirms that the models with sympathetic activity of the subjects and their initial Tsk as predictors were significant for all ROIs considered. Sympathetic activity was a significant independent predictor of Δ-Total in the anterior thigh, posterior thigh and anterior knee but not in the posterior knee, anterior leg, and posterior leg. Baseline Tsk was a significant predictor of Δ-Total in all ROIs except in the posterior knee. The variance explained by the models was low for the posterior knee (R^2^adjusted = 23.0%) but high for the anterior thigh (R^2^adjusted = 63.7%) and the posterior thigh (R^2^adjusted = 64.4%), which correspond to the areas exercised. 

According to our results, Tsk measured by thermography could be used in the future to estimate the level of participation of muscle areas in the exercise; however, the time that elapsed since the end of the exercise should be considered to accurately interpret the thermal results. To clarify this, it should be studied how the initial level of activation of the sympathetic system could influence the response to strength exercise. This would help to understand how incomplete recovery and/or different activation levels of the athlete could lead to different skin thermal responses. Registering the level of sympathetic activation before exercise could be interesting in predicting the response of the athlete to strength training.

We understand that the way to control the intensity of the effort (only the RPE score with values of 6.75 ± 1.18 immediately after exercise and 5.26 ± 1.81 after the 20-min recovery period) could be considered a weakness of our study. In future research, it would be interesting to confirm our results with a stricter control of the physiological effects of the workload performed (for example, lactate test or biopsies) and for other types of strength training with different intensities and durations of the effort.

## 5. Conclusions

Our results confirmed a significant effect of exercise and recovery on Tsk in all ROIs (*p* < 0.05) and strong inverse relationships between sympathetic and parasympathetic activation values in the Tsk. Furthermore, we found that the level of sympathetic activation affects the skin thermal response, being a significant predictor of total Tsk variation only in the exercised muscle areas, and the baseline Tsk could be a significant predictor of total Tsk variation in the all ROIs except in the posterior knee. 

## Figures and Tables

**Table 1:** Tsk on the six ROIs considered and the sympathetic and parasympathetic activation values for the three recorded moments and the results of the repeated measures ANOVA analysis.

Skin Temperatures (°C)	PRE	POST	POST-20	F_(2,36)_	*p*	η²_p_
Anterior Thigh	31.4 ± 0.9	31.3 ± 1.4	32.3 ± 0.8	11.976	<0.001	0.400
Posterior Thigh	31.3 ± 1.0	30.8 ± 1.3	31.5 ± 0.7	6.972	0.003	0.279
Anterior Knee	29.5 ± 1.2	29.0 ± 1.4	30.1 ± 1.0	11.248	<0.001	0.385
Posterior Knee	31.9 ± 0.9	31.1 ± 1.1	31.8 ± 0.9	19.018	<0.001	0.514
Anterior Leg	30.9 ± 1.0	29.7 ± 1.2	30.3 ± 0.7	21.197	<0.001	0.541
Posterior Leg	31.1 ± 1.0	29.5 ± 1.2	29.9 ± 0.8	40.803	<0.001	0.694
Activation levels (0–1)						
Sympathetic	0.56 ± 0.14	0.62 ± 0.20	0.59 ± 0.20	0.970	0.389	0.051
Parasympathetic	0.41 ± 0.14	0.36 ± 0.14	0.39 ± 0.19	0.535	0.590	0.029

**Table 2:** Results of the Spearman correlation analysis (Rho (p)) for sympathetic and parasympathetic activation levels (0 to 1.00) at the three data collection moments (Mean ± SD).

	Sympathetic PRE Mean (0.55 ± 0.14)	Sympathetic POST Mean (0.61 ± 0.19)	Sympathetic POST-20 Mean (0.59 ± 0.20)
Parasympathetic PRE Mean (0.42 ± 0.19)	−0.880 (<0.001) *	−0.372 (0.177)	−0.436 (0.062)
Parasympathetic POST Mean (0.36 ± 0.14)	−0.342 (0.152)	−0.914 (<0.001) *	−0.606 (0.006) *
Parasympathetic POST-20 Mean (0.39 ± 0.19)	−0.268 (0.268)	−0.626 (0.004) *	−0.936 (<0.001) *

* = significant relationship.

**Table 3:** Results of the parametric (Student’s T test) and non-parametric analysis (U of Mann–Whitney) for the Tsk variations after “exercise”, “recovery” and “total” by level of sympathetic activation.

	Lower Activity (°C)	Higher Activity (°C)	Dif (°C)	Test Statistic	*p*	Location Parameter	95% CI		Effect Size	95% CI	
							Lower	Upper		Lower	Upper
Δ-exercise ant_thigh	0.29 (0.90)	−0.29 (1.31)	0.58	1.430 ^a^	0.171	0.769 ^a^	−0.366	1.903	0.657 ^a^	−0.279	1.575
Δ-exercise ant_knee	−0.52 (1.57)	−0.36 (0.89)	0.16	50.00 ^b^	0.720	0.178 ^b^	−0.785	1.380	0.111 ^b^	−0.398	0.568
Δ-exercise ant_leg	−1.19 (0.57)	−1.32 (0.68)	0.13	0.426 ^a^	0.675	0.181 ^a^	−0.715	1.077	0.196 ^a^	−0.710	1.096
Δ-exercise post_thigh	−0.09 (1.03)	−0.61 (0.73)	0.52	1.497 ^a^	0.153	0.558 ^a^	−0.229	1.345	0.688 ^a^	−0.251	1.608
Δ-exercise post_knee	−0.65 (0.55)	−1.18 (0.64)	0.53	1.608 ^a^	0.126	0.477 ^a^	−0.149	1.102	0.739 ^a^	−0.208	1.662
Δ-exercise post_leg	−1.47 (0.49)	−1.73 (1.29)	0.26	1.330 ^a^	0.201	0.443 ^a^	−0.260	1.146	0.611 ^a^	−0.321	1.526
Δ-recovery ant_thigh	1.07 (0.99)	0.52 (1.02)	0.55	56.00 ^b^	0.400	0.339 ^b^	−0.516	1.178	0.244 ^b^	−0.277	0.654
Δ-recovery ant_knee	1.38 (0.81)	0.54 (0.66)	0.84	67.00 ^b^	0.079	0.666 ^b^	−0.110	1.290	0.489 ^b^	0.001	0.789
Δ-recovery ant_leg	0.87 (1.02)	0.30 (0.29)	0.57	56.00 ^b^	0.400	0.497 ^b^	−0.308	1.136	0.244 ^b^	−0.277	0.654
Δ-recovery post_thigh	0.59 (0.61)	0.48 (0.65)	0.13	−0.050 ^a^	0.960	−0.021 ^a^	−0.893	0.852	−0.023 ^a^	−0.923	0.878
Δ-recovery post_knee	0.91 (0.75)	0.59 (0.52)	0.32	54.00 ^b^	0.497	0.238 ^b^	−0.630	0.820	0.200 ^b^	−0.319	0.627
Δ-recovery post_leg	0.48 (0.71)	0.48 (0.65)	0.00	51.00 ^b^	0.661	0.146 ^b^	−0.599	0.725	0.133 ^b^	−0.379	0.583
Δ-total ant_thigh	1.45 (0.75)	0.26 (0.96)	1.19 *	3.466 ^a^	0.003 *	1.039 ^a^	0.406	1.671	1.593 ^a^	0.530	2.621
Δ-total post_thigh	0.33 (0.27)	−0.10 (0.62)	0.44 *	2.107 ^a^	0.050 *	0.538 ^a^	−0.001	1.076	0.968 ^a^	−0.001	1.912
Δ-total ant_knee	0.90 (0.71)	0.15 (0.15)	0.75 *	79.00 ^b^	0.004 *	0.765 ^b^	0.235	1.340	0.756 ^b^	0.424	0.909
Δ-total post_knee	0.09 (0.79)	−0.67 (0.89)	0.76	1.867 ^a^	0.079	0.541 ^a^	−0.070	1.153	0.858 ^a^	−0.098	1.791
Δ-total ant_leg	−0.23 (0.81)	−1.01 (0.50)	0.78	1.473 ^a^	0.159	0.447 ^a^	−0.193	1.087	0.677 ^a^	−0.261	1.596
Δ-total post_leg	−1.05 (0.69)	−1.59 (1.05)	0.54	0.995 ^a^	0.353	0.336 ^a^	−0.406	1.078	0.439 ^a^	−0.480	1.345

Notes: Delta (Δ) values presented as mean (standard deviation) ^(a)^ or median (interquartile range) ^(b)^; Test Statistic: t (from *t* Test) ^(a)^ or W (from Mann-Whitney U test) ^(b)^; Location Parameter: Mean difference and 95% CI ^(a)^ or Hodges-Lehmann estimate and 95% CI ^(b)^; Effect size: Cohen’s d and 95% CI ^(a)^ or Rank-Biserial correlation and 95% CI ^(b)^. * = significant relationship.

**Table 4:** Summary of the results of the regression analysis.

Model	R^2^	Adjusted R^2^	RMSE		F		df1	df2		*p*
Anterior Thigh	0.677	0.637	0.499		16.777 *		2	16		<0.001
		**Unstandardized**	**SE**	**Standardized**	**T**	***p***	**[95% CI]**		**[95% CI]**	
	Anterior Thigh _initial_	−0.550	0.125	−0.628	−4.419 *	<0.001	−0.814		−0.286	
	Sympathetic Activity	−3.182	0.837	−0.540	−3.802 *	0.002	−4.957		−1.408	
**Model**	**R^2^**	**Adjusted R^2^**	**RMSE**		**F**		**df1**	**df2**		***p***
Posterior Thigh	0.683	0.644	0.362		17.271 *		2	16		<0.001
		**Unstandardized**	**SE**	**Standardized**	**T**	***P***	**[95% CI]**		**[95% CI]**	
	Posterior Thigh _initial_	−0.451	0.084	−0.752	−5.343 *	<0.001	−0.814		−0.286	
	Sympathetic Activity	−1.358	0.607	−0.315	−2.237 *	0.040	−2.645		−0.071	
**Model**	**R^2^**	**Adjusted R^2^**	**RMSE**		**F**		**df1**	**df2**		***p***
Anterior Knee	0.522	0.463	0.521		8.750 *		2	16		0.003
		**Unstandardized**	**SE**	**Standardized**	**T**	***p***	**[95% CI]**		**[95% CI]**	
	Anterior Knee _initial_	−0.281	0.105	−0.464	−2.685 *	0.016	−0.502		−0.059	
	Sympathetic Activity	−2.688	0.875	−0.531	−3.071 *	0.007	−4.543		−0.832	
**Model**	**R^2^**	**Adjusted R^2^**	**RMSE**		**F**		**df1**	**df2**		***p***
Posterior Knee	0.315	0.230	0.591		3.682 *		2	16		0.048
		**Unstandardized**	**SE**	**Standardized**	**T**	***p***	**[95% CI]**		**[95% CI]**	
	Posterior Knee _initial_	−0.288	0.155	−0.386	−1.859	0.082	−0.615		0.040	
	Sympathetic Activity	−1.779	0.996	−0.371	−1.787	0.093	−3.889		0.332	
**Model**	**R^2^**	**Adjusted R^2^**	**RMSE**		**F**		**df1**	**df2**		***p***
Anterior Leg	0.537	0.479	0.492		9.281 *		2	16		0.002
		**Unstandardized**	**SE**	**Standardized**	**T**	***p***	**[95% CI]**		**[95% CI]**	
	Anterior Leg _initial_	−0.494	0.120	−0.705	−4.134 *	<0.001	−0.748		−0.241	
	Sympathetic Activity	−0.726	0.828	−0.150	−0.878	0.393	−2.481		−1.028	
**Model**	**R^2^**	**Adjusted R^2^**	**RMSE**		**F**		**df1**	**df2**		***p***
Posterior Leg	0.402	0.328	0.626		5.384 *		2	16		0.016
		**Unstandardized**	**SE**	**Standardized**	**T**	***p***	**[95% CI]**		**[95% CI]**	
	Posterior Leg _initial_	−0.466	0.150	−0.609	−3.111 *	0.007	−0.784		−0.149	
	Sympathetic Activity	−0.571	1.064	−0.105	−0.537	0.599	−2.826		1.683	

* = significant relationship; SE = Standard error.

## Data Availability

Not application.
